# Case of Tubal Fœtation

**Published:** 1850-09

**Authors:** C. C. Warner

**Affiliations:** Summerville, Wisconsin


### ARTICLE X.

*Case of Tubal Fœtation.*—By C. C. Warner, M. D., of Summerville, Wisconsin.

Mrs. L. C. was married April 4th, 1849 (in her sixteenth year), and the 25th day of January (Friday) was delivered of a healthy male child.

Her husband was with her at the time she was delivered, and remained until the Wednesday following, and left her, but returned Feb. 16th. He staid until the 25th, went away, and has not returned since, making in all just one month from the time her husband was last with her. She did well after her confinement, and in ten days was able to be about the house, and do some light work. While he was at home the last time (Feb. 21st.), I was called to see her, and found her laboring under inflammatory absorption of the gums, and a slight Fever. Calomel and Jalap aa. grs. 10 were administered, and a gargle of Nitre, ʒiij. dissolved in a pint of Barley Water, and subsequently Blue Mass from 3 to 5 grains every evening with gargles of Cinchon. and Alum, until the bowels became active, when the morbid affection of the gums subsided. Her general health, however, did not improve, but declined into a chlorotic condition, with a bad cough and frothy expectoration, frequently to the amount of a pint in twenty-four hours. March 5th, was again called, and found her in the above condition. She had nursed her child up to the present time, though for the last week had had but little for him. I ordered him taken from her, and prescribed an expectorant of Scilla, ℈ij.; Ant. Tartras., gr. j.; Camph. Tinct. of Opium, ʒij., and water and sugar sufficient to form a syrup of ℥iij.; dose 3 drops every six hours, and wine and Carb. Ferri as much as she would bear. She gradually improved, and in two weeks was able to sit up most of the time, walk about the house by taking hold of something to steady her, and ride a short distance. During the whole time since I was last called, she had had a very craving appetite, ate freely of nearly every dish that could be prepared by her mother, and all of her neighbors (and those not a few), and nothing of the kind ever seemed to hurt her, though she vomited frequently in the morning. I came to the conclusion that she was again pregnant; although she had previously informed me that she had had no discharge from the vagina, to her knowledge, of any kind, since the first week after she was delivered of her child. April 10th. Pulse 100 in a minute, and quick, but easily compressed, and had been so for the last three days.

Changed from the wine and iron to Hydriodate of Potassa gr. ij., three times a-day, and Squill and Digitalis aa. gr. j. in in pill. She continued to improve. April 18th, rode to her sister's (a distance 1½ miles), and on the morning of the 19th was taken with pain in her bowels (as she expressed herself) and vomiting; was carried home; pain and vomiting continued at intervals through the day; bowels moved twice. Sulph. Morphine was prescribed, sufficient to relieve the pain. I informed her friends that she was in labor, and that abortion would probably take place. But fearing the almost certain consequence, death, from the debilitated chlorotic condition of the system, I continued the morphine, gr. ¼, combined with Quinine, gr. j. every six hours for a tonic, and Calomel and Ipecac. aa. gr. j. every four hours, with a mustard cataplasm over the epigastric region to allay the irritability of the stomach, and perfect rest, that, if possible, the present symptoms might be allayed.

Tuesday, 23d. Dr. Wheeler was called in; thought the difficulty might arise from a disordered condition of the liver, and recommended the Morphine and Quinine continued, and Extract Cicuta for an alterative. This was continued through the day, but to no effect.

24th. Pain and vomiting continued as before; was again put upon the former prescription, but all gave only temporary relief. During the last 36 hours, was almost constantly calling for food, but it, on being taking into the stomach, was soon thrown up again.

She died on the morning of the 25th, just three months to a day from the time she was confined, and two from the time her husband last left her. In the evening of the same day, a post mortem examination was made by myseif in presence of two other gentlemen. The mucous membrane of the uterus was found somewhat congested, and a membranous substance in the womb, though separate from the mucous surface, and resembling in appearance a small clot. The left ovary was found to contain two corpora lutea, one a very little larger than the other, and in the fallopian tube, about half-way from the fimbriated extremity to the womb, was a fœtus of about two months growth. The place where it was lodged was enlarged into a sac, and somewhat congested. The right ovary and fallopian tube were natural in appearance, and contained no marks of any kind. The above is a description of the parts as they appeared to the naked eye. Dr. Blanchard, of Delavan, afterwards saw the preparation which I have now in my possession, and agreed with me in the above description of the parts.

